# Towards Novel Amino Acid-Base Contacts in Gene Regulatory Proteins: AraR – A Case Study

**DOI:** 10.1371/journal.pone.0111802

**Published:** 2014-11-03

**Authors:** Isabel Lopes Correia, Irina Saraiva Franco, Isabel de Sá-Nogueira

**Affiliations:** 1 Departamento de Ciências da Vida (DCV), Centro de Recursos Microbiológicos (CREM), Faculdade de Ciências e Tecnologia (FCT-UNL), Caparica, Portugal; 2 Instituto Tecnologia Química e Biológica (ITQB-UNL), Oeiras, Portugal; Universität Stuttgart, Germany

## Abstract

AraR is a transcription factor involved in the regulation of carbon catabolism in *Bacillus subtilis*. This regulator belongs to the vast GntR family of helix-turn-helix (HTH) bacterial metabolite-responsive transcription factors. In this study, AraR-DNA specific interactions were analysed by an *in vitro* missing-contact probing and validated using an *in vivo* model. We show that amino acid E30 of AraR, a highly conserved residue in GntR regulators, is indirectly responsible for the specificity of amino acid-base contacts, and that by mutating this residue it will be possible to achieve new specificities towards DNA contacts. The results highlight the importance in DNA recognition and binding of highly conserved residues across certain families of transcription factors that are located in the DNA-binding domain but not predicted to specifically contact bases on the DNA. These new findings not only contribute to a more detailed comprehension of AraR-operator interactions, but may also be useful for the establishment of a framework of rules governing protein-DNA recognition.

## Introduction

Protein–DNA binding is a process fundamental to life as it masters many genetic activities such as transcription, recombination, DNA replication and repair. The specific interaction between transcription factors and their cognate DNA sites is critical for regulation of gene expression in cells. Understanding how these different proteins are able to find and bind selectively to only one, or just a small number, specific sequence(s) out of the millions of nucleotides present in a genome is a major goal of molecular biology. The recognition principles of protein–DNA interfaces are guided by the complex interplay of noncovalent interactions [1], [2], [3], [4]. In general, DNA recognition follows two paradigms, direct and indirect readout. In the case of direct readout, proteins form contacts such as, hydrogen bonds and van der Waals contacts, mainly in the major, and to a lesser extent also the minor, groove of the DNA to the edges of the base pairs to probe the DNA sequence [1], [2], [3], [4]. Indirect readout occurs through protein contacts to the DNA that depend on base pairs that are not directly contacted by the protein in which the sequence-dependent deformability or structural differences between DNA molecules contribute to their discrimination. A DNA–protein “recognition code”, although of great utility in molecular biology, remains elusive and improbable. While it is clear that a single recognition code does not exist there is some evidence for the existence of a degenerated code whereby one group of bases displays tendency to interact with a certain group of amino acids [4], [5], [6]. In recent years, researchers have addressed this issue by strengthening a comprehensive framework of the rules governing protein–DNA interactions. Different strategies have been described for the construction of Zinc-fingers (ZFs) and TAL (transcription activator-like) proteins with new binding specificities [7], [8]. Nevertheless, there is not a simple one-to-one correspondence between protein and DNA sequences, thus direct readout alone is insufficient to justify the specificities of protein-DNA interactions.

AraR is a homodimeric transcription factor involved in the regulation of carbon catabolism in *Bacillus subtilis*. The protein displays a chimeric organization, consisting of two functional domains with different phylogenetic origins [9], [10]: a small N-terminal DNA-binding domain (DBD) comprising a winged helix–turn–helix (HTH) motif belonging to the GntR family of transcriptional regulators [11] and a larger C-terminal domain homologous to that of the GalR/LacI family of bacterial regulators and sugar-binding proteins [12]. Recently, the three-dimensional crystal structure of the AraR C-terminal domain [13] and the DNA-binding domain [14] were independently solved. AraR typifies one of the GntR-subfamilies of proteins (reviewed in [15]). The GntR superfamily is one of the largest groups of HTH bacterial metabolite-responsive transcription factors (Pfam family: PF00392; Prosite Family PS50949) and GntR-like regulators are widespread in bacteria and are known to control many fundamental cellular processes, such as primary metabolism, motility, development, antibiotic production, antibiotic resistance, plasmid transfer and virulence (reviewed in [15]).

**Figure 1:**
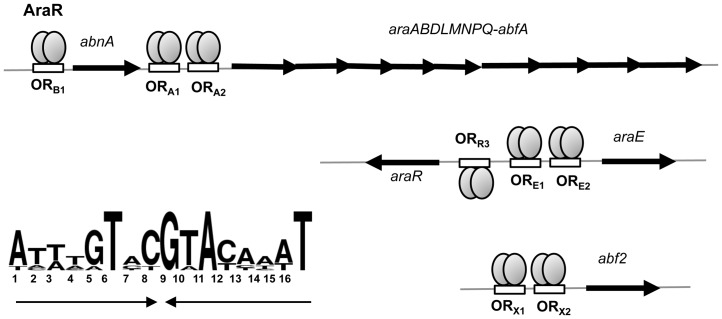
The arabinose (*ara*) regulon comprises thirteen genes located in three different regions of the chromosome. The genes are represented as black arrows pointing at the direction of transcription. The AraR repressor, in the absence of the effector molecule - arabinose - binds to palindromic sequences (At(T/A)tGTaCGTAcaa(A/T)T consensus depicted, bottom left) found in the promoter region of the *ara* genes. The AraR protein is shown as a dimer. The eight AraR boxes are represented as white rectangles. Binding to the different operators may either be cooperative or uncooperative.

The control in gene expression exerted by AraR is modulated by the presence of the inducer L-arabinose. Binding of AraR to L-arabinose leads to induction of expression of the *ara* regulon (Figure 1), which is composed of at least thirteen genes. The products of these genes include the regulator itself, extracellular and intracellular catabolic enzymes involved in the degradation of arabinose-, galactose- and xylose-containing polysaccharides, uptake of these sugars into the cell and further catabolism of L-arabinose and arabinose oligomers [9], [16], [17], [18]. In the absence of inducer, AraR recognizes and binds at least eight palindromic operator sequences (*ara* boxes), located in the five known arabinose-inducible promoters (Figure 1). Three of these promoters contain two *ara* boxes: the promoter of the *araABDLMNPQ-abfA* operon (boxes OR_A1_ and OR_A2_), of *araE* (OR_E1_ and OR_E2_) and of *abf2* (OR_X1_ and OR_X2_). In the cases of the genes *araR* and *abnA*, a single box is present (OR_R3_ and OR_B1_) (Figure 1). AraR binding to the promoters displaying two boxes is cooperative, requiring in phase and properly spaced operators, and involves the formation of a small loop in the DNA. These two mechanistically diverse modes of action of AraR result in distinct levels of transcriptional regulation, as cooperative binding to two *ara* boxes results in a high level of repression while interaction with a single operator allows a more flexible control [10], [18], [19].

Previous studies have mapped the functional domains of AraR and characterized the C-terminal region involved in effector binding and dimerization [20]. Moreover, guided by molecular modelling we identified amino acids potentially involved in DNA binding and the effect of their substitution revealed key residues necessary for the DNA binding and regulatory activity *in vivo* and *in vitro* [21]. In addition, important bases for AraR-DNA interactions in both arms of the palindromic operator sequences were also identified [21]. In this work we studied AraR-DNA specific interactions using methodologies designed to detect direct or indirect interactions between the atoms/residues of the interacting partners, both *in vitro* and *in vivo*. AraR mutant proteins displaying a moderate effect in AraR-DNA interaction and single point mutations in the operator DNA leading to partial derepression of gene expression were probed. The results obtained provide valuable information concerning the specific interaction of AraR-DNA and insights into the binding of GntR regulators in general.

## Materials and Methods

### Strains and growth conditions

*Escherichia coli* DH5α (Gibco BRL) was used as host for routine molecular cloning work. *E. coli* strains were grown in LB [22] medium and the antibiotics ampicillin (100 µg ml^−1^) and tetracycline (12 µg ml^−1^) were added when appropriated. *B. subtilis* strains used in this study (Table 1) were grown in liquid in LB or C-minimal medium [23] and chloramphenicol (5 mg ml^−1^), kanamycin (10 mg ml^−1^) or erythromycin (1 mg ml^−1^) were added when appropriate. The *B. subtilis* and *E. coli* cells were transformed as described previously [7]. The Amy phenotype was tested by detection of starch hydrolysis on tryptose blood agar base medium (Difco) plates, containing 1% (w/v) of potato starch, with an I_2_–KI solution as described previously [9]. The Thr phenotype was determined by growth on Spizizen minimal medium [24] supplemented with 2% (w/v) of glucose, 0.2% (w/v) potassium glutamate, 3 mM MgSO_4_, and 2% (w/v) agar.

**Table 1:** *Bacillus subtilis* strains used in this work.

Strain	Genotype	Source^a^^,^ b
168T^+^	*Prototroph*	F. E. Young
IQB 215	*ΔaraR::km*	[9]
IQB 568	*ΔaraR::km araAB′-lacZ ermΔamyE::araR* E30A *cat*	[21]
IQB 571	*ΔaraR::km araAB′-lacZ ermΔamyE::araR* Y5F *cat*	[21]
IQB 761	*ΔaraR::km ΔamyE::araR cat*	pLS30→IQB215
IQB 778	*ΔaraR::km ΔamyE::araR* E30A *cat*	pMI35→IQB215
IQB 774	*ΔaraR::km ΔamyE::araR* Y5F *cat*	pMI36→IQB215
IQB 771	*ΔaraR::km ΔamyE::araR cat ΔthrC::OR_A1A2_*_ WT_ *-lacZ erm*	pMI37→IQB761
IQB 790	*ΔaraR::km ΔamyE::araR cat ΔthrC:: OR_A1_ T_6_→G -lacZ erm*	pMI48→IQB761
IQB 772	*ΔaraR::km ΔamyE::araR cat ΔthrC:: OR_A1_ T_16_→G -lacZ erm*	pMI46→IQB761
IQB 773	*ΔaraR::km ΔamyE::araR cat ΔthrC:: OR_A1_ A_1_→C -lacZ erm*	pMI46→IQB761
IQB 779	*ΔaraR::km ΔamyE::araR* E30A *cat ΔthrC::OR_A1A2_*_ WT_ *-lacZ erm*	pMI37→IQB778
IQB 798	*ΔaraR::km ΔamyE::araR* E30A *cat ΔthrC:: OR_A1_ T_6_→G -lacZ erm*	pMI48→IQB778
IQB 796	*ΔaraR::km ΔamyE::araR* E30A *cat ΔthrC:: OR_A1_ T_16_→G -lacZ erm*	pMI45→IQB778
IQB 797	*ΔaraR::km ΔamyE::araR* E30A *cat ΔthrC:: OR_A1_ A_1_→C -lacZ erm*	pMI46→IQB778
IQB 775	*ΔaraR::km ΔamyE::araR* Y5F *cat ΔthrC::OR_A1A2_*_ WT_ *-lacZ erm*	pMI37→IQB774
IQB 791	*ΔaraR::km ΔamyE::araR* Y5F *cat ΔthrC:: OR_A1_ T_6_→G -lacZ erm*	pMI48→IQB774
IQB 792	*ΔaraR::km ΔamyE::araR* Y5F *cat ΔthrC:: OR_A1_ T_16_→G -lacZ erm*	pMI45→IQB774
IQB 793	*ΔaraR::km ΔamyE::araR* Y5F *cat ΔthrC:: OR_A1_ A_1_→C -lacZ erm*	pMI46→IQB774
IQB 926	*ΔaraR::km ΔamyE::araR cat ΔthrC::OR_x1x2_*_ WT_ *-lacZ erm*	pMI64→IQB761
IQB 927	*ΔaraR::km ΔamyE::araR cat ΔthrC:: OR_x1_ T_6_→G -lacZ erm*	pMI64→IQB761
IQB 928	*ΔaraR::km ΔamyE::araR* E30A *cat ΔthrC::OR_x1x2_*_ WT_ *-lacZ erm*	pMI63→IQB778
IQB 929	*ΔaraR::km ΔamyE::araR* E30A *cat ΔthrC:: OR_x1_ T_6_→G -lacZ erm*	pMI63→IQB778

a The arrows indicate transformation and point from donor DNA to recipient strain.

b Transformation was carried out with linearized DNA.

### DNA manipulation and construction of plasmids

DNA manipulations were carried out as described by Sambrook *et al.* [25]. Restriction enzymes were purchased from MBI Fermentas and used according to the manufacturer's instructions. DNA was eluted from agarose gels with GFX gel band purification kit (Amersham Pharmacia Biotech). DNA sequencing was performed with ABI PRIS BigDye Terminator Ready Reaction Cycle Sequencing kit (Applied Biosystems). PCR amplifications were done using high-fidelity Phusion DNA polymerase (Finnzymes) and the resulting products purified by QIAquick PCR purification kit (Qiagen).

For the construction of plasmids pMI35 and pMI36, bearing substitutions E30A and Y5F, respectively, the mutated *araR* alleles were amplified by PCR with primers ARA1 and ARA73 (Table 2), using as template chromosomal DNA from strains IQB568 and IQB571 [21], respectively. The PCR products were digested with EcoRI-BamHI (or EcoRI-BglII) and independently subcloned in the respective pLS30 sites [20]. The obtained plasmids were then digested with ScaI, which allows the occurrence of a double crossover recombination event at *amyE* locus of the *B. subtilis* chromosome (Table 1).

**Table 2:** Oligonucleotides used in this work.

Primer	Sequences (5′→3′)	Complementary sequence
ARA1	(−39) TAAGGGTAACTATTGCCG (−22)	pSN32 (fwd)
ARA73	(+77) CTTCCACAGTAGTTCACC (+60)	pSN32 (rev)
ARA87	(−207) AAAATAGCGGATTACGGCATCG (−186)	*abf2* (fwd)
ARA262	(−37) GATTGACAGTATAATAGTCAATTAC (−13)	*araABDLMNPQ-abfA*
ARA263	(+90) CCCTTTCTCATAAAATAAAACGC (+68)	*araABDLMNPQ-abfA*
ARA542	(−75) TAAATACAGACGTACAAATAT (−54)	OR_X1_ T_6_→G (fwd)
ARA541	(−54) ATATTTGTACGTCTGTATTTA (−75)	OR_X1_ T_6_→G (rev)

Plasmids pMI37, pMI45, pMI46 and pMI48, contain respectively, the wild-type of the *araABDLMNPQ-abfA* operon promoter and the same promoter bearing mutation OR_A1_ (T_16_→G), OR_A1_ (A_1_→C) or OR_A1_ (T_6_→G), respectively, fused to a *lacZ* gene. These plasmids were constructed by insertion of the 204-bp BamHI-EcoRI DNA fragment from pLM32 [10], pLM67, pLM68, pLM65 [21], respectively, into the same sites of pDG1663 [26], to generate an *OR_A1A2_ -lacZ* fusion, suitable for a double crossover recombination event at *thrC* locus of the *B. subtilis* chromosome (Table 1). To create *abf2* promoter - *lacZ* fusions the wild-type and the mutated *abf2* promoter, OR_X1_ (T_6_→G) were inserted into the vector pDG1663 to yield plasmids pMI64 and pMI63, respectively. For construction of pMI64, a 291-bp EcoRI-BamHI DNA fragment from pRIT1 [18] bearing the *abf2* wild-type promoter was subcloned into those sites of pDG1663. Mutagenesis of the *abf2* promoter, OR_X1_ (T_6_→G), was achieved by PCR overlap extension, regions immediately upstream and downstream of mutagenesis target region were amplified in two independent PCR experiments, using primers ARA87 and ARA542 (PCR1) using as template chromosomal DNA of *B. subtilis* 168T^+^ and primers ARA541 and ARA73 (PCR2), using pRIT1 as template. The products were joined by overlapping PCR, with primers ARA87 and ARA73 (Table 2), and the resulting fragment was digested with BamHI and EcoRI and cloned into pDG1663 BamHI-EcoRI, yielding pMI63.

### β-Galactosidase assays

*B. subtilis* strains were grown in C-minimal medium supplemented with 1% (w/v) casein hydrolysate in the presence and absence of 0.4% (w/v) L-arabinose, as previously reported [9]. Samples of cell culture were collected and analysed 2 h after the addition of L-arabinose, β-Galactosidase activity was measured using the substrate *p*-nitrophenyl-β-D-galactoside (ONPG) and expressed in Miller units, the ratio of β-galactosidase activity in the presence and absence of inducer was taken as a measure of AraR repression in the analysed strains (Repression Index) as described previously [9].

### Electrophoretic mobility shift assay (EMSA)

DNA fragments carrying the operator sequences OR_A1A2_ wild-type and mutants OR_A1_ A_1_→C, G_5_→T, T_6_→G, and T_16_→G were amplified by PCR, with primers ARA262 and ARA263, using plasmids pLM51, pLM61, pLM62 and pLM58 [21], respectively, as template. Overexpression and protein purification of the AraR wild-type and mutant variants (Y5F and E30A) were performed as described previously [20].

The assays were performed as described in Franco *et al.* [21], DNA fragments were radiolabelled with [γ-^32^P] dATP using T4 Polynucleotide Kinase. The protein-DNA binding reaction was carried out in a volume of 10 µl containing 12 mM HEPES-KOH pH 7.6, 10 mM MgCl_2_, 0.5% (w/v) BSA, 1 mM DTT, 10% Glycerol (v/v), 200 mM NaCl, 4 mM Na_2_HPO_4_, 4 mM NaH_2_PO_4_, 0.4 mM EDTA, a 200-fold molar excess of competitor DNA (polydIdC), 1 nM of labelled DNA and increasing concentrations of wild-type or mutant AraR proteins, and incubated at room temperature for 30 min. The reaction mixtures were then submitted to electrophoresis on a native 8% polyacrylamide gel containing Tris-glycine buffer (25 mM Tris, 200 mM glycine, pH 8.9) and run at 100 V for ∼1 h. Gels were *vacuum* dried and exposed on a Phosphorimager screen before analysis with a Molecular Dynamics Storm 860 Imager and ImageQuant version 5.0.

The determination of the dissociation constants, *K*_d_ values, was obtained using the GraphPad Prism software and the “one site total binding” model, following the equation Y = B_max_^.^X/(*K*_d_+X)+NS^.^X, with X = AraR concentration, Y = bound protein, B_max_ is the maximum specific binding and NS is the slope of nonspecific binding. Concentrations of AraR were determined assuming a pure dimeric protein. Differences between *K*_d_ were analyzed by Mann Whitney U test using SPSS software, P<0.05 was considered as the level of statistical significance. *The* value 0.057 (Table 3) was considered moderate evidence against the null hypothesis [H0: On average there is no difference in binding affinity of the two DNA fragments (mutant DNA fragment *vs* wild-type DNA fragment)]. The association constant (*K*_ass_) is calculated from *K*_d_ = 1/*K*_ass_, and the Gibbs free energy (Δ*G*°) by ΔG° = −*RT* ln *K*_ass_.

**Table 3:** Thermodynamic parameters of AraR-DNA interaction reactions.

	Dissociation constant *K*_d_ (×10^−8^) (M)^a^								
	DNA fragment *araABDLMNPQ-abfA* promoter								
Protein	*OR_A1_*WT	*OR_A1_T_6_→G*	*p* b	*OR_A1_T_16_→G*	*p* b	*OR_A1_A_1_→C*	*p* b	*OR_A1_G_5_→T*	*p* b
AraR WT	2.7±0.5	5.6±0.4	0.016	11.0±3.3	0.016	7.4±1.6	0.038	34.5±9.5	0.008
AraR E30A	19.8±5.3	**25.4±6.7**	0.412	34.7±6.4	0.057	67.5±12.9	0.029	74.8±18.7	0.057
AraR Y5F	27.4±3.2	47.6±9.0	0.016	**35.5±11.1**	0.214	**33.1±7.6**	0.343	>200	0.016

	Δ*G*° of the AraR-DNA association reactions^c^								
	DNA fragment *araABDLMNPQ-abfA* promoter								
Protein	*OR_A1_*WT	*OR_A1_T_6_→G*	ΔΔ*G*°^d^	*OR_A1_T_16_→G*	ΔΔ*G*°^d^	*OR_A1_A_1_→C*	ΔΔ*G*°^d^	*OR_A1_G_5_→T*	ΔΔ*G*°^d^
AraR WT	−10.18	−9.76	0.42	−9.36	0.82	−9.59	0.59	−8.69	1.48
AraR E30A	−9.02	**−8.87**	**0.15**	−8.69	0.32	−8.30	0.72	−8.24	0.78
AraR Y5F	−8.83	−8.50	0.32	**−8.68**	**0.15**	**−8.72**	**0.11**	ND	ND

a Dissociation constant (*K*_d_) of binding of AraR and AraR mutants (Y5F, E30A) to a wild-type *araABDLMNPQ-abfA* promoter (WT) and mutants (OR_A1_ T_6_→G, T_16_→G, A_1_→C, G_5_→T) calculated by densitometric quantification of the bands corresponding to free DNA and protein–DNA complex by EMSA (see Materials and Methods). The values represent the average and standard deviation of at least three (three to seven) independent assays, with an intrinsic error <31%.

b *p-value* (Mann Whitney U test) for each pairwise comparison, mutant DNA fragment *vs* wild-type DNA fragment. Mutated operators that did not show statistical significant variation (*p*<0.05) when compared to the wild-type operator are highlighted in bold.

c Gibbs free energy is calculated from the equilibrium association constant *K*_ass_ (see Materials and Methods).

d Variation of Gibbs free energy = [Δ*G*°mutant−Δ*G*°wild-type]. ND – not determined.

## Results

### Probing amino acid-base contacts *in vitro*

**Figure 2:**
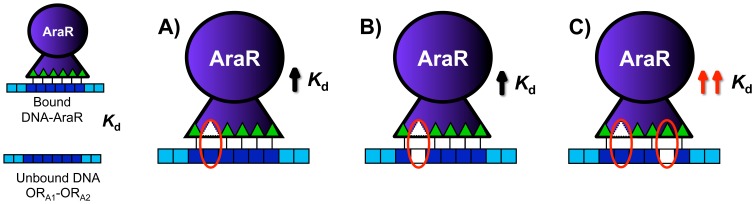
Rationale of the *in vitro* experiment. Schematic representation of the AraR protein in dark grey, and DNA fragment in light grey comprising one operator sequence in dark grey. Each base is represented by a square. Amino acids in contact with the DNA are depicted as triangles. Open triangles indicate mutated amino acids. Open squares represent mutated base. Arrows denote increase in *K*_d_. A) A certain mutation in an amino acid leads to an increase in *K*_d_ for the wild-type operator as consequence of a specific interaction that was lost; B) any DNA position normally contacted by the altered amino acid may be mutated with little or no effect; C) any DNA position not involved in contacts by the altered amino acid when mutated leads to a cumulative increase in *K*_d_.

In a previous study aimed at understanding the specific properties of the interaction AraR-operator sequences, we substituted amino acids, in or near the winged-HTH motif, which according to the model were predicted to contact DNA [20], [21], and the effects of these substitutions on the ability of AraR to function *in vivo* and on the DNA-binding affinities *in vitro* were determined [20], [21]. Conversely, mutational analysis of the AraR-binding sites was used to determine the base-specific requirements for transcriptional regulation *in vivo* and DNA binding *in vitro*. These experiments showed that specific AraR residues and operator bases are crucial to achieve a high level of regulatory activity, while others display variable contributions to DNA binding. In order to characterize in detail the AraR-DNA specific interaction we used the loss-of-contact approach [27]. In this study we initially used an *in vitro* missing-contact probing [28], [29] using electrophoretic mobility shift assay (EMSA) to determine the binding affinities of AraR and mutant proteins to a DNA fragment bearing the promoter of the metabolic operon with two operators (OR_A1_-OR_A2_) and the same fragment comprising single base pair substitutions in the OR_A1_ box (AATTGTTCGTACAAAT). The rationale of these experiments was as following: a certain amino acid alteration leads to an increase in *K*_d_ for the wild-type operator (Figure 2A); if this increment is the consequence of a lost direct or indirect interaction between that particular amino acid and a specific base, when we use a DNA fragment with a substitution in that particular base we expect no major effect in the *K*_d_, when compared to the wild-type DNA, because a particular contact had already been lost and quantified (Figure 2B); in contrast, if the amino acid exchanged is not involved in contacts with the specific mutated base we will expect an additional increase in *K*_d_ (Figure 2C).

**Figure 3:**
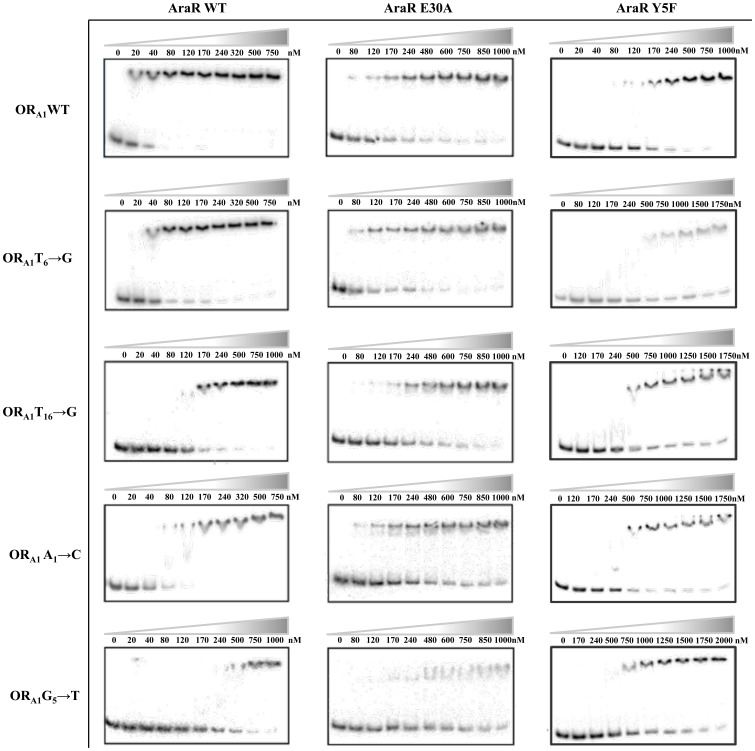
Analysis of operator mutations on AraR–DNA affinity *in vitro* by EMSA. AraR wild type left column; AraR E30A middle column; and AraR Y5F mutant right column. The indicated amounts of AraR protein were used in the binding reactions, AraR was incubated with the 5′-end labelled probe (1 nM) bearing the wild-type or mutant operators OR_A1_-OR_A2_ and the protein-DNA complexes were resolved on native 8% polyacrylamide gels. The mutation in each DNA fragment is depicted.

This methodology, in addition to indicating residues directly involved in contacts with bases may also reveal amino acids whose presence is important to maintain the overall structural arrangement of the protein even though they do not directly contact bases in the DNA. For the experiments we chose AraR mutant proteins, AraR Y5F and AraR E30A, which displayed a moderate effect in AraR-DNA interaction both *in vivo* and *in vitro*, and base pair substitutions leading to partial derepression *in vivo*, A_1_→C, G_5_→T, T_16_→G and T_6_→G [21]. The results of the EMSA are summarized in Figure 3 and the calculated *K*_d_ values are shown in Table 3. The AraR wild-type protein showed a statistical significant decrease in the affinities for a DNA fragment bearing the promoter of the metabolic operon with two operators (OR_A1_-OR_A2_), when we compared the wild-type DNA fragment to the same fragment bearing mutations in the OR_A1_ box. Previously, we have shown that binding of AraR to OR_A1_-OR_A2_ is cooperative and a single point mutation in either OR_A1_ and OR_A2_ causes an almost complete loss of AraR regulation in vivo [10], [19]. Similarly, in vitro a single-point mutation in OR_A1_ reduces dramatically the apparent affinity of AraR for the second operator OR_A2_ [10].

The AraR E30A protein displayed a decrease in the affinity for all mutated operators except for the T_6_→G operator (Table 3). In fact, AraR E30A showed no additional significant decrease in the affinity, relative to the wild-type operator, when the T_6_→G operator mutant was used (Figure 3 and Table 3). As T_6_ in OR_A1_ is important for protein binding [21], and the T_6_→G mutation did not reduce the binding affinity of AraR E30A, this suggests that this operator substitution did not further affect the loss of contact of AraR E30A. The *K*_d_ of the mutant AraR Y5F for the operator mutations tested revealed a significant a reduction in the affinity compared to the wild-type for G_5_→T and T_6_→G, but not for A_1_→C or T_16_→G (Figure 3 and Table 3). This could indicate that Y5 might be relevant for the contact of AraR with T_16_ and A_1_ of OR_A1_. Because these nucleotides are located in opposite positions in the palindromic sequence of the operator, this observation suggests that Y5 of one monomer is important for the interaction with A_1_, while the other contacts T_16_. However, the crystal structure of the AraR-DNA binding domain bound to OR_A1_ [14] showed Y5 interacting with the DNA backbone near nucleotide T_6_ (see below).

In summary, the results obtained *in vitro* suggest that AraR residue E30 may play an important role in the interaction of the protein with the T_6_ nucleotide.

### *In vivo* validation of protein-DNA interactions

**Figure 4:**
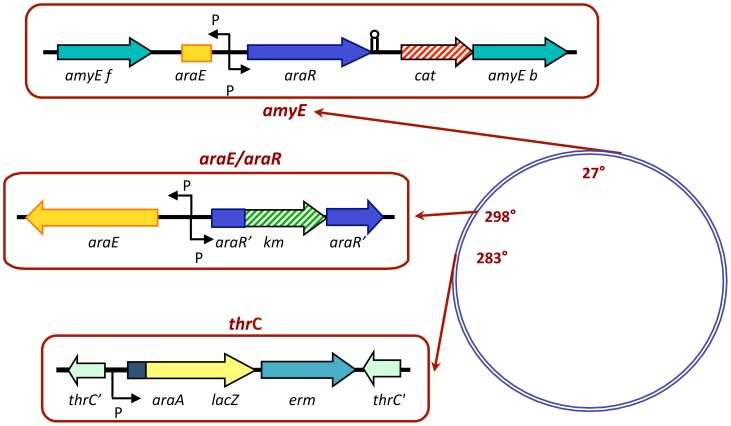
Genetic organization of the reporter *B. subtilis* strains. The circle illustrates the *B. subtilis* chromosome and the location of the *amyE*, *araE/araR*, and *thrC* loci indicated in degrees. The construction containing the wild-type or mutant *araR* alleles placed at the *amyE locus* is represented in the top left. The *araR-null* genetic background is depicted in middle left. The regulatory activity exerted by the *araR* alleles over the wild-type or mutant *araA* promoter sequences is measured by a promoter *lacZ* fusion placed at the *thrC locus* (bottom left).

Since the experimental conditions used to derive *K*_d_ values bear little resemblance to intracellular situations, the *in vitro* results were confirmed by *in vivo* assays. For this, we constructed *B. subtilis* strains in order to confront the different *araR* alleles and mutant DNA operator sequences in the same cell. The different *araR* alleles were ectopically introduced at the *amyE locus* of an *araR* null mutant background. Additionally a transcriptional fusion between the *araA* promoter, carrying the OR_A1_-OR_A2_ operators, and the *E. coli lacZ* gene, was generated and ectopically introduced at the *B. subtilis thrC locus* (Figure 4). This genetic system allows us to measure the regulatory activity of the native and mutant proteins over distinct promoters (wild-type and mutated) fused to the *lacZ* reporter gene by determination of the levels of accumulated β-galactosidase. In previous studies we have shown that in these conditions the cellular level of both mutant proteins AraR E30A and AraR Y5F is comparable to that seen with wild-type AraR, ruling out the possibility of deregulation originated by degradation of the repressor [21]. The results of the confrontation of the different *araR* alleles and the various promoters in the series of strains constructed are summarized in Table 4.

**Table 4:** Regulatory activity of the wild-type AraR protein and mutants E30 and Y5 over an *araA-lacZ* promoter fusion (wild-type and mutated variants).

			β-Galactosidase activities (MU)^a^		
*araR allele*	Strain	*araA′-lacZ*	−ara	+ara	*R.I.* b
*araRwt*	IQB771	*OR_A1A2_ wt*	17.5±2.2	1032.44±134.3	59.4±7.4
	IQB790	*OR_A1_ T_6_→G*	99.5±12.8	1308.79±113.2	13.4±1.7
	IQB772	*OR_A1_ T_16_→G*	453.9±15.3	1815.72±133.2	4.0±0.2
	IQB773	*OR_A1_ A_1_→C*	84.4±5.37	1205.67±72.9	14.3±0.0
*E30A*	IQB779	*OR_A1A2_ wt*	431.8±38.2	1490.9±104.3	3.5±0.1
	IQB798	*OR_A1_ T_6_→G*	82.3±7.1	773.8±74.5	9.4±0.8
	IQB796	*OR_A1_ T_16_→G*	1583.2±61.8	1429.2±170.9	0.9±0.1
	IQB797	*OR_A1_ A_1_→C*	1413.0±189.9	1429.3±187.3	1.0±0.0
*Y5F*	IQB775	*OR_A1A2_ wt*	172.8±0.56	1462.9±33.5	8.5±0.2
	IQB791	*OR_A1_ T_6_→G*	1670.2±24.2	1409.2±34.1	0.8±0.0
	IQB792	*OR_A1_ T_16_→G*	1890.7±63.5	1454.7±112.0	0.8±0.1
	IQB793	*OR_A1_ A_1_→C*	1362.4±30.3	1497.3±121.7	1.1±0.1

a β-Galactosidase activities of the *B. subtilis* strains grown in the absence (−ara) or presence (+ara) of arabinose. Values represent the average and standard deviation of at least three independent experiments, each assayed in duplicate. MU Miller units.

b R.I. (Repression Index) indicates the regulatory activity, calculated as the ratio between values obtained in the presence and in the absence of inducer.

The analysis of repression index of the wild-type AraR with the different promoter fragments showed a decrease in the regulatory activity when a mutated box OR_A1_ was used, compared to the wild-type OR_A1A2_. The mutation OR_A1_ T_16_→G displayed the higher deregulation, while OR_A1_ A_1_→C and T_6_→G exhibited similar less drastic effects. These results are comparable to those obtained in the *in vitro* assays (Table 3). The dissociation constant of the mutant Y5F suggested that this amino acid might interact with two nucleotides in the operator sequence, T_16_ and A_1_ (Table 3). However, the *in vivo* analysis does not corroborate the hypothesis (Table 4), as mutations at position T_16_ and A_1_ have a drastic effect in the regulatory activity of mutant Y5F (IQB792 and IQB793; Table 4). The *in vivo* results are in agreement with the results of the crystal structure of the AraR-DNA binding domain bound to OR_A1_ [14] that revealed Y5 interacting with the DNA backbone near nucleotide T_6_, thus this residue is not involved in direct or indirect contact with T_16_ and A_1_ (discussed below).

The EMSA assays indicated that residue E30 could be relevant for the interaction of the AraR protein with the T_6_ nucleotide (Table 3), although both the N-terminal AraR model [21] and the N-terminal AraR-OR_A1_ structure [14] suggest non-specific contacts of E30 to the DNA backbone (discussed below). This observation was supported by the *in vivo* data because the regulatory activity of mutant AraR E30A over the mutant OR_A1_ T_6_→G-*lacZ* promoter fusion is 2.7-fold higher (strain IQB798, Table 4) than that observed for the wild type promoter OR_A1A2_WT-*lacZ* (strain IQB779, Table 4). Furthermore, the lower level of expression observed in the strain bearing the mutant AraR E30A and the mutant OR_A1_ T_6_→G-*lacZ* promoter fusion (strain IQB798, Table 4), both in the presence and absence of inducer, compared to that obtained in the strain harbouring the wild-type AraR regulator and the mutant OR_A1_ T_6_→G-*lacZ* promoter fusion (strain IQB790, Table 4) suggests a stronger interaction of the E30A protein towards the mutated DNA operator.

Overall the *in vivo* results highlight the importance of amino acid E30 in the regulatory activity AraR and in the contact of the protein with the nucleotide T_6_ in OR_A1_.

### Residue E30 is important for the AraR regulatory activity in distinct promoters

As T_6_ is a well-conserved nucleotide in the consensus signature of the AraR DNA binding site, present in all AraR operators characterized so far (Figure 1), to establish that E30 is an important amino acid for the AraR contact to the thymine at position 6 we assayed this effect in the context of a different promoter. The *abf2* gene is regulated by cooperative binding of AraR to two in-phase operators OR_X1X2_ similarly to that observed in the arabinose metabolic operon promoter ([18]; Figure 1). Thus, using the same strategy the wild-type OR_X1_ (ATACATACGTACAAAT) and mutant OR_X1_T_6_→G *abf2′-lacZ* fusions were constructed and introduced at the *B. subtilis thrC locus*.

The analysis of the regulatory index exerted by the native AraR in the strain IQB927 showed no effect of *OR_X1_T_6_→G* mutation when compared to the wild-type promoter (strain IQB926, Table 5). On the other hand, mutant AraR E30A leads to a complete loss of the regulation of the wild-type *abf2′-lacZ* promoter fusion *abf2*, showing once again the importance of this amino acid in the regulatory mechanism of this transcription factor. Nevertheless, the confrontation of the mutant E30A with mutation T_6_→G (strain IQB929, Table 5) leads to an increase in the regulatory activity when compared to the wild-type promoter (strain IQB928, Table 5). Therefore, the T_6_→G single nucleotide change partially suppresses the loss of regulation caused by the E30A amino acid substitution pointing out that E30 is an important amino acid for the AraR contact to the thymine at position 6 of both operator sequences OR_A1_ and OR_X1_.

**Table 5:** Regulatory activity of the wild-type AraR protein and mutant E30A over an *abf2-lacZ* promoter fusion (wild-type and mutated variant).

			β-Galactosidase activities (MU)^a^		
*araR allele*	Strain	*abf2′-lacZ*	−ara	+ara	*R.I.* b
*araR wt*	IQB926	*OR_X1X2_ wt*	7.2±1.1	119.4±16.6	16.7±0.2
	IQB927	*OR_X1_T_6_→G*	27.2±3.08	450.4±66.9	16.6±1.7
*E30A*	IQB928	*OR_X1X2_ wt*	155.8±12.9	133.3±14.7	0.9±0.1
	IQB929	*OR_X1_T_6_→G*	148.2±21.8	336.7±40.5	2.3±0.2

a β-Galactosidase activities of the *B. subtilis* strains grown in the absence (−ara) or presence (+ara) of arabinose. Values represent the average and standard deviation of at least three independent experiments, each assayed in duplicate. MU Miller units.

b R.I. (Repression Index) indicates the regulatory activity, calculated as the ratio between values obtained in the presence and in the absence of inducer.

## Discussion

The sequence-specificity of DNA recognition by proteins should be viewed in a complete framework. At the atomic level the specificity of DNA-binding proteins is mainly accomplished through direct hydrogen bond and hydrophobic interactions between specific amino acid side chains and functional groups of nucleotide bases in the major and minor groove [1], [2], [3], [4], [30]. Nevertheless these direct or water-mediated hydrogen bonds are insufficient to completely explain the specificity of many DNA-binding proteins. In addition to the chemical complementarity between protein and DNA atoms, it is required a structural complementarity along the networking surfaces of the protein and DNA molecules [31]. The use of genetic methods to identify amino acid base pair contacts in a specific protein-DNA complex is a complementary approach to the X-ray diffraction and to two-dimensional nuclear magnetic resonance spectroscopic (2D NMR) analyses. Furthermore, the construction and analysis of single amino acid substitutions is the only method to determine the apparent binding free energy contribution and the apparent specificity free energy contribution of an amino acid-base pair contact [27 and references therein].

The GntR family members, in general, possess a DNA binding at the N-terminus of the protein and an effector-binding and/or oligomerisation domain at the C-terminus (Pfam family: PF00392; Prosite Family PS50949; [15]). The DNA-binding domain is conserved throughout the GntR family, consisting of a 3-helical bundle core with a small beta-sheet (wing), winged-HTH motif. Despite the vast number of GntR family members sequences deposited in databases there are only a few crystal structures available to examine in detail structure/function relationships. AraR is a transcription factor that typifies one of the sub-families of the GntR group, and recently the three-dimensional crystal structure of the AraR C-terminal domain [13] and the DNA-binding domain [14] were separately and independently determined. In this work, AraR was used to characterize specific interactions with the DNA by an *in vitro* missing-contact probing and posterior validation *in vivo*. In the *in vitro* a fragment

The results obtained *in vitro* with the AraR wild-type protein correlate well with those previously obtained in *in vivo* experiments [19], except for the mutation G_5_→T that showed a more accentuated decrease in the affinity measured *in vitro* than the loss of regulation observed *in vivo* [21]. Moreover, the data obtained *in vivo* in this study with the AraR wild-type protein are consistent with those previously observed *in vivo* using a different genetic system [21]. Although, The *in vitro* EMSA analysis using AraR mutant Y5F and the different DNA fragments bearing point mutations in the OR_A1_ operator suggested that residue Y5 could be important for protein contacts with two nucleotides in opposite sites of the operator palindromic sequence, T_16_ and A_1_ (Table 3), however the *in vivo* results do not corroborate this hypothesis (Table 4). The *in vivo* results validate the data of the crystal structure of the AraR DNA-binding domain in complex with two different operators, OR_A1_ and OR_R3_, showing specific contacts with DNA [14]. In fact, Y5 is not involved in direct or indirect contact with these nucleotides because it interacts with the DNA backbone near nucleotide T_6_. The analysis of the *in vitro* interaction between mutant AraR E30A with the mutant DNA fragments A_1_→C, T_16_→G and G_5_→T revealed a decrease in affinity when compared to the wild-type DNA indicating that residue E30 is not indirectly involved in contacts with the mutated bases. These mutated nucleotides are highly conserved across all AraR operators characterized so far [21], and accordingly to the AraR-OR_A1_ structure involved in the interaction with the protein. The opposite nucleotides of A_1_ and T_16_ are contacted by the same amino acid, G62, through an acetated or water-mediated interaction, respectively, but from different monomers, while G_5_ establishes a direct contact with amino acid R41 [14]. Surprisingly, the *in vitro* interaction studies with mutant T_6_→G displayed no decrease in the affinity of the mutant AraR E30A suggesting that residue E30 could be indirectly involved in contacts with T_6_. Furthermore, *in vivo* analysis performed with two distinct promoters showed that mutation T_6_→G partially suppresses the effect of substitution E30A in AraR improving its regulatory activity. In both strains bearing a *lacZ* fusion to different promoters an increase in the regulatory activity of the mutant E30A is observed (IQB798 Table 4 and IQB929 Table 5). Thus, the presence of an alanine at position 30 seems to have positive contribution to the interaction of the mutant OR_A1_ T_6_→G with the protein.

**Figure 5:**
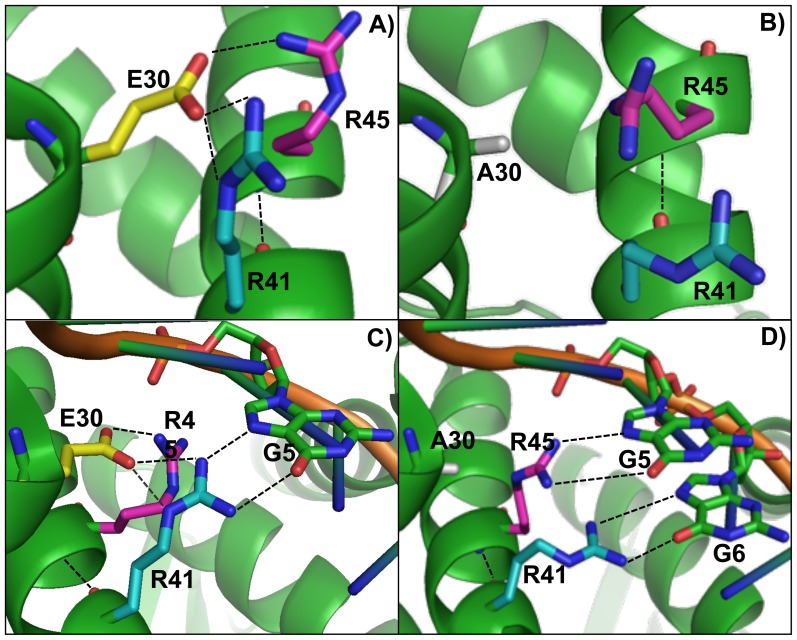
Model for the interaction between mutant AraR E30 and the mutant operator. Native AraR structure displaying interactions between: **A**) residues E30, R41 and R45 and **B**) residues (E30, R41 and R45), and R41 and guanine 5 (OR_A1_). Mutant AraR E30A displaying possible interactions between: **C**) residues E30A, R41 and R45 and **D**) residues (R41, R45) and guanine 5 and 6 (OR_A1_ T_6_→G). E30 (yellow), A30 (grey), R41 (light blue), R45 (purple) and direct hydrogen bonds (side chain or protein-DNA) are shown in dashed lines. The structures were drawn using PyMOL (http://pymol.sourceforge.net/) and the data of the structure of the AraR N-terminal domain in complex with OR_A1_ (PDB access no. 4EGY; [14]).

The E30 residue is highly conserved in the GntR-family proteins, and the corresponding residue in FadR, E34, was shown to contact the DNA backbone [32], [33]. The FadR-DNA structure indicates that E34 also contacts nearby amino acids, contributing presumably to the stabilization of residues that interact specifically with the DNA bases. Similarly, both the N-terminal AraR model and the N-terminal AraR-OR_A1_ structure suggest non-specific contacts of E30 to the DNA backbone [14], [21], and indicate possible interactions with R41 and R45 [14], [21]; and Figure 5A). The core of HTH motif is comprised by two α-helices, H2 and H3, spaced by a short four-residues turn (T) in between. In AraR E30 belongs to H2, the stabilizing helix, while R41 and 45 to H3, the recognition helix. The angle between H2 and H3 is typically of 120°, however it can vary between 100° and 150° [34]. Since E30 interacts with R41 and R45, this interaction is crucial to settle the geometry and spatial arrangement of H2 and H3, and protein docking on DNA by the recognition helix, H3 (Figure 5A and B). The role of the E30 is not only the interaction with the DNA but is also to limit the rotation of the recognition helix. In the E30A mutant, R41 and R45 are no longer interacting with E30, moreover this alanine substitution impairs the contacts of this residue with the DNA backbone (Figure 5C). As a result, the regulatory activity of the mutant protein decreases in the presence of the wild-type *ara* operon promoter, which does not occur in the presence of mutant OR_A1_ T_6_→G promoter as a consequence of a spatial orientation of H2 and H3 (Table 4). On the other hand, enrichment of the operator DNA with another guanine, T_6_→G, could lead to a significant alteration in DNA conformation. In fact, the exocyclic 2-amino groups of the guanines are crucial elements in DNA structure and recognition, as they are known to exert a substantial influence on DNA bending, flexibility and intrinsic curvature [35], [36], [37], [38]. Therefore if the functional groups in the protein do not correctly juxtapose with those in the DNA, protein-DNA complex stability is impaired, which seems to be the case of the wild-type AraR interaction with the mutated operator T_6_→G. An amino acid not directly involved in contacts with bases, such as E30, placed within or adjacent to the DNA binding domain can therefore indirectly affect the affinity of the protein to DNA by properly modulating the protein conformation, allowing a correct alignment between the functional groups of the protein and the DNA.

Although there is no ‘recognition code’ between amino acids and nucleotides, they possess some preferential interactions, for instance arginines are known to interact favourably with guanines [4], [5], [6]. Thus, we propose that the effect observed *in vivo* of the recovery of regulation in the double mutant E30A OR_A1_ T_6_→G is due to the loss of interaction between E30, and R41 or R45, which results in a conformational change that allows a proper arrangement between the functional groups of the protein and the new operator DNA composition. R41 and R45 became free to establish new interactions with the nucleotides, not only the G at position 5, but also with the new G at position 6 (Figure 5D). Thus, the E30A mutation results in a better contact of the latter residues (R41 or R45) with G5 and the mutated G6 adjusting to the new DNA sequence, as observed by the increased regulatory activity of the mutant protein in the presence of the mutated operators (OR_A1_ and OR_X2_) when compared to the native protein (Table 4 and Table 5).

Our results provide information beyond the pairwise analysis, the data highlight and demonstrate that residues that are not involved in specific interactions with nucleotides, but act as linker residues by positioning other amino acids in the correct 3D context of a nucleoprotein complex, can be as important for the protein-DNA interaction as residues making direct contact with DNA bases, and have a crucial role in the modulation of DNA recognition. Furthermore, we show that by manipulating these residues it is possible to redesign the specificity of protein–DNA interactions.
